# Validation of the *Pneumocystis* pneumonia score in haematology patients with acute respiratory failure

**DOI:** 10.1186/s12890-020-01279-4

**Published:** 2020-09-05

**Authors:** Ryoung-Eun Ko, Jongmin Lee, Soo Jin Na, Na Ri Jeong, Seon Woo Kim, Kyeongman Jeon

**Affiliations:** 1 Department of Critical Care Medicine, Samsung Medical Center, Sungkyunkwan University School of Medicine, 81 Irwon-ro, Gangnam-gu, Seoul, 06351 Republic of Korea; 2 Division of Pulmonary, Allergy and Critical Care Medicine, Department of Internal Medicine, Seoul St Mary’s Hospital, Collage of Medicine, The Catholic University of Korea, Seoul, Republic of Korea; 3 Biostatistics and Clinical Epidemiology Center, Samsung Medical Center, Sungkyunkwan University School of Medicine, Seoul, Republic of Korea; 4 Division of Pulmonary and Critical Care Medicine, Department of Medicine, Samsung Medical Center, Sungkyunkwan University School of Medicine, 81 Irwon-ro, Gangnam-gu, Seoul, 06351 Republic of Korea

**Keywords:** *Pneumocystis* pneumonia, Invasive fungal infection, Immunocompromised, Respiratory failure

## Abstract

**Background:**

*Pneumocystis* pneumonia (PCP) is an important cause of acute respiratory failure (ARF) in immunocompromised patients, yet no actual clinical tool suitably identifies patients at risk. Recently, a multivariable prediction model has been proposed for haematology patients with ARF requiring intensive care unit (ICU) admission to assess the risk of PCP (PCP score). However, it has not yet been validated externally.

**Methods:**

To validate the PCP score, a retrospective cohort study was conducted in two large designated haematology centres in Korea. One-hundred and forty haematology patients with ARF were admitted to ICU. They underwent aetiologic evaluations between July 2016 and June 2019. The predictive ability of the score was assessed with the receiver operating characteristic (ROC) curve analysis for both the discrimination and calibration of the score.

**Results:**

Among the 141 patients, 13 (9.2%) were finally diagnosed of PCP. Although the median of PCP score in PCP group was higher than in non-PCP group (3.0 [interquartile range 0.0–4.0] vs. 2.0 [0.5–4.0]), the difference was not statistically significant (*P* = 0.679). The area under the ROC curve of the PCP score in our cohort was 0.535 (95% CI, 0.449–0.620), indicating no discriminatory ability. When using a cut-off of 3.0 the score, the result was 38.5% (95% CI, 13.9–68.4) sensitive and 7.03% (95% CI, 61.6–78.1) specific. The negative predictive value was 58.8% and positive predictive value was 59.8% for a 10% prevalence of PCP.

**Conclusions:**

In this study, the PCP score was not useful to predict the risk of PCP in haematology patients with ARF. Further prospective validation studies are needed to validate the score’s use in routine clinical practice for the early diagnosis of PCP in haematology patients.

## Background

*Pneumocystis* pneumonia (PCP), a pulmonary infection caused by *Pneumocystis jirovecii*, is an important cause of acute respiratory failure (ARF) in immunocompromised patients [1–5]. PCP is most commonly associated with the human immunodeficiency virus (HIV) infection. However, the development of highly active antiretroviral therapy and effective prophylaxis against PCP have reduced its prevalence and the mortality rate in HIV-positive patients [6, 7]. Consequently, more attention is placed on other immunocompromised states [8–10].

Haematologic malignancies are the most common underlying conditions associated with the development of PCP in HIV-negative patients [11, 12]. Compared to HIV-positive patients who follow a more indolent course [13, 14], haematology patients with PCP present with abrupt-onset hypoxemic respiratory failure, and more often require mechanical ventilation [13–15]. In addition, delays in anti-PCP treatment are associated with poor outcomes [13, 16]. However, inappropriate use of TMP/SMX should be avoided not only prevent drug related side effects including granulocytopenia, hepatotoxicity, and nephrotoxicity, but also prevent delay of inaccurate diagnosis or resistant strains. Nonetheless, the confirmative diagnosis of PCP with bronchoalveolar lavage (BAL) would be challenging in haematology patients with hypoxemic respiratory failure, making it difficult to perform bronchoscopy [10, 14]. Therefore, a clinical tool that rapidly identifies patients at risk of PCP (in whom empiric treatment is warranted), should be developed and consequently avoiding delays in anti-PCP treatment [17].

Recently, Azoulay et al. introduced a multivariable prediction model to assess the risk of PCP (PCP score) for haematology patients with ARF requiring ICU admission and they reported a good performance of the PCP score [18]. However, there have been no external validations of the prediction model with other cohorts. In this study, we then assessed the performance of the PCP score in haematology patients from two large designated haematology centres in Korea.

## Methods

We retrospectively reviewed the medical records of all consecutive haematology patients admitted in the medical intensive care unit (ICU) for respiratory failure at Samsung Medical Center (a 1989-bed, university-affiliated, tertiary referral hospital in Seoul, South Korea) and Seoul St. Mary’s Hospital (a 1369-bed, university-affiliated, tertiary referral hospital in Seoul, South Korea) between July 2016 and June 2019. The Institutional Review Boards of each participating hospital with patient records approved the present study and the informed consent was waived because of the non-interventional nature of this research. All patient records and data were anonymised and coded prior to analysis.

### Study population

**Fig. 1:**
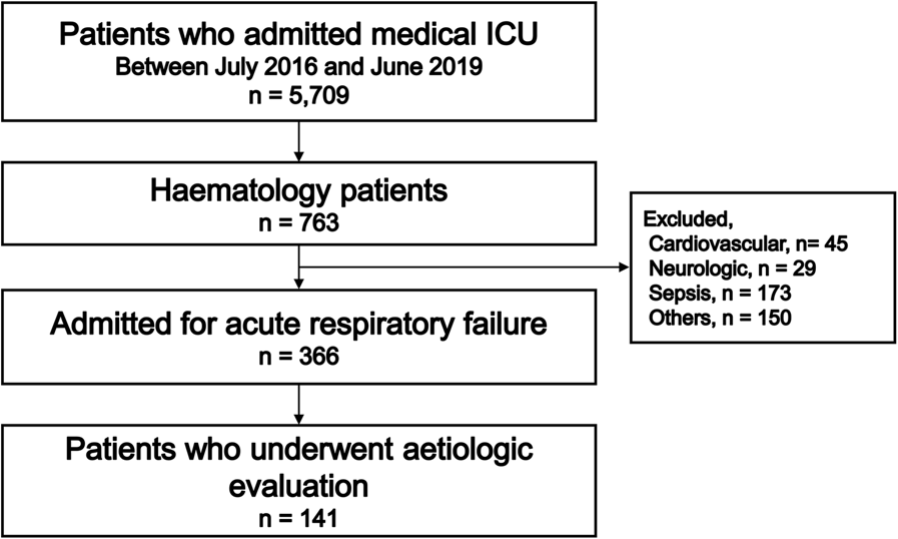
Study flow diagram. ICU, intensive care unit

All consecutive haematology patients older than 20 years who were admitted to the medical ICU for ARF were screened for inclusion. Patients were included if they received BAL with or without a transbronchial lung biopsy (TBLB) for aetiologic explorations, and a results of microbiological identification of *Pneumocystis jirovecii* in BAL fluid or lung tissue were included (Fig. 1). Patients were excluded if they had a positive HIV antibody test. For cases with multiple admissions for ARF during the study period, only the first ICU admission was evaluated.

### *Pneumocystis* pneumonia score

Azoulay et al. developed a PCP score for haematology patients with ARF using a cohort with PCP prevalence of 11.2% (149 of 1330), in which PCP was confirmed by identification of *P. jirovecii* cysts or trophozoites in BAL fluid or induced sputum [18]. The PCP score uses parameters: age, lymphoproliferative disease, anti-PCP prophylaxis, day between respiratory symptom onset and ICU admission, shock at ICU admission, and chest X-ray results (showing pleural effusion or not, gotten at ICU admission). The PCP score ranged from - 6 to 8.5, and higher scores indicated more possibility of PCP and the cut-off value of the PCP score was found to be 3.0 from the validation cohort.

### Data collection

Data extracted from the medical records include general demographic information, underlying haematologic disease and medications received during the previous month. Furthermore, PCP prophylaxis, initial presentation of symptoms, vital signs/organ supports at ICU admission and chest radiographic findings. We also collected laboratory data such the white blood cell count, albumin, C-reactive protein, procalcitonin, lactate dehydrogenase, and haemoglobin. Severity of illness was assessed using the Sequential Organ Failure Assessment (SOFA) score [19]. The data included integral components of the PCP score [18].

The diagnosis of PCP was based on the identification of the organism in BAL fluid or lung tissue obtained by TBLB. BAL fluid samples were stained using Gram and Ziehl-Neelsen methods and then cultured for bacteria, mycobacteria and fungi. Microbiological identification of *P. jirovecii* was confirmed by documenting the organism with Wright Giemsa or Gram-Weigert stain or the cyst with Gomori methenamine silver or calcofluor white stain [1], which is the same methods with the previous study [18].

### Statistical analysis

Data was presented as medians and interquartile ranges (IQR) for continuous variables and as numbers and percentages for categorical variables. The baseline and clinical characteristics on ICU admission were compared between the PCP group and the non-PCP group using the Mann-Whitney U test (for continuous variables) and the Pearson’s chi-square test or Fisher exact test (for categorical variables).

To determine the sample size needed for the validation of our prediction model [20], we based our calculations on the test characteristics of the PCP score. For a ROC curve area of 0.8, a power (type II error) of 80% and an α (type I error) of 0.05, the sample size required for our study was 103 (8 from the positive group and 95 from the negative group). This was calculated using MedCalc statistical software.

The predictive ability of PCP score was assessed with ROC curve analysis for both the discrimination (via the C index) and calibration (using Hosmer–Lemeshow statistics) of the score [21]. Univariable logistic regression analysis was performed to estimate the odds ratios (ORs) of each variable in the PCP score. The ORs of each variable were reported with their 95% confidence intervals (CIs). Sensitivity, specificity, positive and negative likelihood ratios (LR+ and LR-, respectively) were calculated for the PCP score. All variables were analysed using R Statistical Software (Version 3.2.5; R Foundation for Statistical Computing, Vienna, Austria).

## Results

### Study population

For this study, 141 haematology cases were admitted to the ICU due to ARF. They all underwent BAL and/or TBLB for aetiologic explorations. Amongst all cases, 13 (9.2%) were diagnosed of PCP. Baseline characteristics of the patients are summarised in Table 1. The median age of the patients was 58.0 (IQR 49.0–65.0) years, and 90 (63.8%) patients were male. There were 73 (51.8%) lymphoproliferative diseased cases and the most common haematological disorder was acute/chronic myeloid leukaemia (*n* = 44, 31.2%) followed by non-Hodgkin lymphoma (*n* = 50, 33.6%). Amongst all cases, 42 (29.8%) received an allogenic stem cell transplantation and 19 (13.5%) received an autologous stem cell transplantation. Sixty-three (44.7%) patients received systemic steroid treatment with a median prednisolone-equivalent dose of 44.5 (35.1–51.3) mg/day. Thirty-two (22.7%) cases received T-cell immunosuppressors and six (4.3%) cases received immune checkpoint inhibitors. However, only 30 (21.3%) cases received anti-PCP prophylaxis.

**Table 1:** Baseline characteristics of haematology patients with acute respiratory failure

	PCP (*n* = 13)	No-PCP (*n* = 128)	*P* value
Age, year	54.0 (49.0–64.0)	59.0 (49.0–65.0)	0.585
Sex, male	12 (92.3)	78 (60.9)	0.052
Underlying disease			
Myeloid disease			0.896
Acute/chronic myeloid leukaemia	4 (30.8)	40 (31.2)	
Myelodysplastic syndrome	1 (7.7)	16 (12.5)	
Other myeloid disease	1 (7.7)	6 (4.7)	
Lymphoproliferative disease			
Acute/ chronic lymphocytic leukaemia	2 (15.4)	21 (16.4)	
Non-Hodgkin lymphoma	4 (30.8)	26 (20.3)	
Myeloma	0 (0.0)	10 (7.8)	
Hodgkin lymphoma	0 (0.0)	4 (3.1)	
Others	1 (7.7)	5 (3.9)	
Stem cell transplantation			0.492
Allogenic	2 (15.4)	40 (31.2)	
Autologous	11 (7.7)	8 (6.2)	
Oncologic malignancy	2 (15.4)	4 (3.1)	0.172
Steroid user			0.740
High-dose steroids	2 (15.4)	32 (25.0)	
More than 3 months	2 (15.4)	11 (8.6)	
Within 1 month	2 (15.4)	14 (10.9)	
Dose of steroid over the duration, mg (prednisolone equivalent)	2155 (350–4000)	2750 (900–5303)	0.661
Duration, days	48.0 (12.0–163.0)	62.0 (25.0–165.0)	0.566
Specific drugs			0.084
T-cell immunosuppressors	1 (14.3)	32 (43.2)	
Immune checkpoint inhibitor	2 (28.6)	4 (5.4)	
Bactrim prophylaxis^*^	0 (0.0)	30 (23.4)	0.107

Values are given as median (interquartile range) or number (percentage)

*PCP Pneumocystis* pneumonia

*Defined as prescribed according to the patient (or relatives); adherence was not assessed

The clinical characteristics on ICU admission are displayed in Table 2. The median duration from symptoms to ICU admission was 4.0 (2.0–8.0) days. Amongst all cases, 137 (91.9%) had hypoxaemia requiring mechanical ventilation (*n* = 88, 59.1%) or high-flow nasal cannula support (*n* = 49, 32.9%). Thirty-nine (26.2%) cases needed vasopressor support, five (3.4%) needed renal replacement therapy and one needed extracorporeal membrane oxygenation (0.7%). The median initial SOFA score on ICU admission was 7.0 (4.0–9.0).

**Table 2:** Clinical characteristics on ICU admission

	PCP (*n* = 13)	No-PCP (*n* = 128)	*P* value
Symptom to ICU admission, day	10.0 (2.0–19.0)	6.0 (2.0–12.5)	0.594
Severity score at ICU admission			
Initial SOFA	7.0 (5.0–11.0)	8.0 (5.0–10.0)	0.929
Presented symptom			
Cough	6 (46.2)	57 (44.5)	1.000
Sputum	1 (7.7)	39 (30.5)	0.158
Myalgia	3 (23.1)	55 (43.0)	0.274
Neutropenia within 1 week	6 (46.2)	42 (32.8)	0.509
Vital sign at ICU admission			
SpO_2_, %	93.0 (88.0–97.0)	95.0 (91.0–97.5)	0.326
Respiratory rate, breaths/min	25.0 (24.0–32.0)	27.0 (20.0–32.0)	0.954
Heart rate, beats/min	111.0 (98.0–131.0)	115.0 (100.5–136.0)	0.620
Body temperature, °C	37.4 (36.6–38.4)	37.5 (36.8–38.3)	0.482
Glasgow coma scale	15.0 (14.0–15.0)	15.0 (13.0–15.0)	0.596
PaO_2_/FiO_2_ ratio	136.0 (100.8–191.0)	143.0 (104.5–202.8)	0.948
Laboratory test			
WBC, ×10^3^/μl	3.0 (1.4–6.7)	5.6 (2.4–13.4)	0.151
ALC, ×10^3^/μl	0.3 (0.2–1.2)	0.7 (0.2–1.6)	0.628
Albumin, g/dL	2.9 (2.4–3.3)	2.9 (2.7–3.3)	0.783
C-reactive protein, mg/dL	20.2 (8.7–22.9)	13.7 (7.1–21.3)	0.279
Procalcitonin, ng/mL	0.5 (0.1–4.3)	0.7 (0.3–2.6)	0.490
Lactate dehydrogenase, IU/L	646.0 (564.0–728.0)	943.5 (613.0–1642.0)	0.064
Haemoglobin, g/dL	9.9 (9.0–10.5)	9.1 (8.3–10.4)	0.354
Initial organ support at ICU admission			
Ventilation support			0.651
Mechanical ventilation	8 (61.5)	79 (58.5)	
High-flow nasal cannula	4 (30.8)	45 (33.3)	
Non-invasive ventilation	0 (0.0)	1 (0.8)	
Shock	5 (38.5)	37 (28.9)	0.916
Requiring renal replacement therapy	0 (0.0)	9 (7.0)	0.695
Chest radiography findings			
Focal or diffuse alveolar pattern	3 (23.1)	40 (31.2)	1.000
Focal or diffuse interstitial pattern	6 (46.2)	60 (46.9)	1.000
Focal or diffuse alveolar-interstitial pattern	6 (46.2)	56 (43.8)	1.000
Pleural effusion	3 (23.1)	41 (32.0)	0.727

Values are given as median (interquartile range) or number (percentage)

*ICU* intensive care unit, *SOFA* sequential organ failure assessment, *WBC* white blood cell, *ALC* absolute lymphocyte count, *PCP Pneumocystis* pneumonia

### Performance of the PCP score

**Fig. 2:**
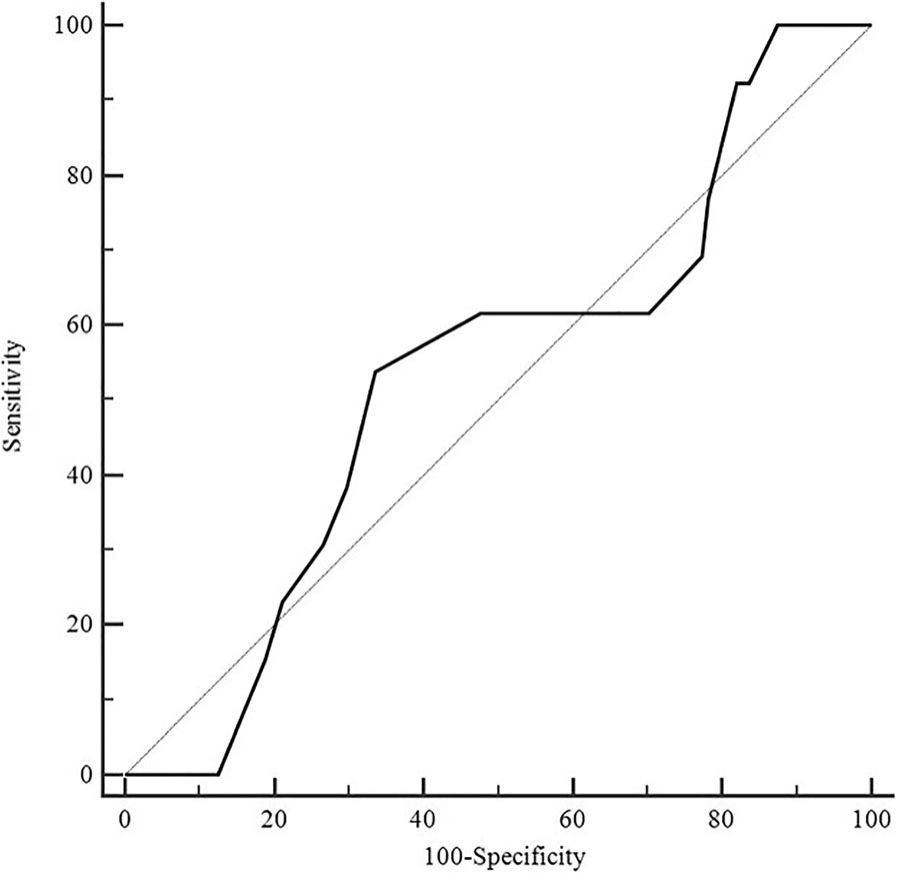
The receiver operating characteristic (ROC) curves for the *Pneumocystis* pneumonia score. The area under the ROC curve was 0.535 (95% CI, 0.449–0.620)

**Fig. 3:**
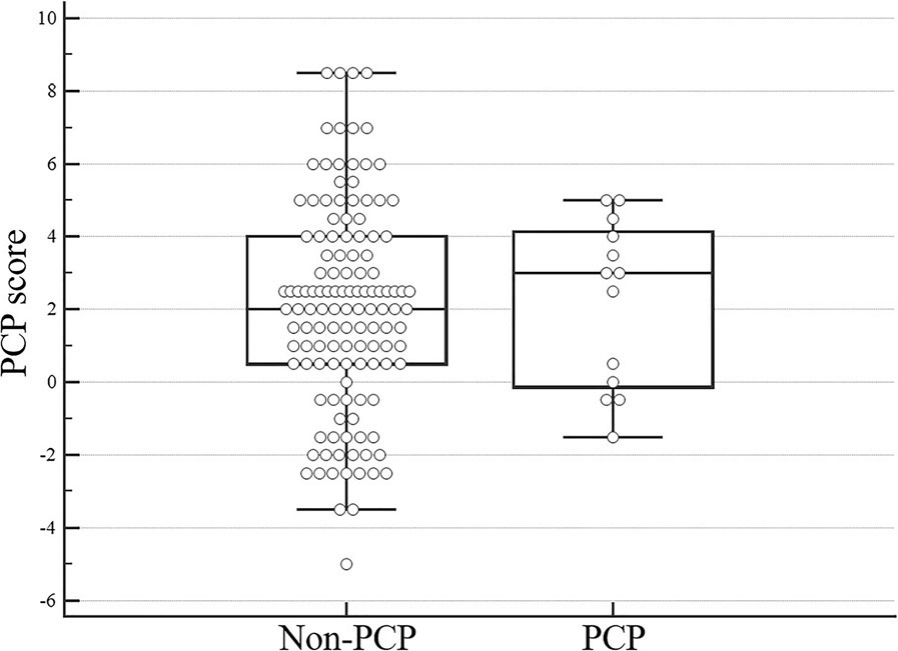
Comparison of the PCP score between PCP and non-PCP groups. The median of PCP scores are 3.0 (0.0–4.0) in PCP group and 2.0 (0.5–4.0) in non-PCP group, which was not statistically significant (*P* = 0.679). Data are presented as medians and interquartile ranges. PCP, *Pneumocystis* pneumonia

The area under the ROC curve was 0.535 (95% CI, 0.449–0.620), indicating no discriminatory ability in our haematology patients with ARF (Table 3, Fig. 2). Comparisons of PCP scores between PCP and non-PCP groups are illustrated in Fig. 3, but no significant difference was observed (*P* = 0.679). Of 13 patients, 7 (53.8%) received a PCP score of 3.0 or higher. When using a cut-off PCP score value of 3.0, a sensitivity of 38.5% (95% CI, 13.9–68.4) and a specificity of 7.03% (95% CI, 61.6–78.1) were obtained. The negative predictive value was 58.8% and the positive predictive value was 59.8% for a 10% prevalence of PCP. Performances of other cut-off values are reported in Table 4.

**Table 3:** Performances of the PCP score

**Area under the ROC curve**	
AUC (95% CI)	0.535 (0.449–0.620)
Z statistics	0.425
**Youden index**	
Youden index J	0.2025
Associated criterion	> 2.5
Sensitivity	53.82
Specificity	66.41
**Using a cut-off 3.0**	
Sensitivity, 95% CI	38.5% (13.9–68.4)
Specificity, 95% CI	70.3% (61.6–78.1)
Negative predictive value (for a PCP prevalence of 10%)	58.8%
Positive predictive value (for a PCP prevalence of 10%)	59.8%
Positive likelihood ratio	1.30
Negative likelihood ratio	0.88

Values are given as median (interquartile range)

*PCP Pneumocystis* pneumonia, *ROC* Receiver Operating Characteristic, *AUC* area under the curve, *CI* confidence interval

**Table 4:** Performances of different cut-off points of the PCP score

Pneumocystis score	Sensitivity	95% CI	Specificity	95% CI	LR+	LR-
≥ − 5	100.00	75.3–100.0	0.00	0.0–2.8	1.00	
> − 2	100.00	75.3–100.0	12.50	7.3–19.5	1.14	0.00
> − 1.5	92.31	64.0–99.8	16.41	10.5–24.0	1.10	0.47
> − 1	92.31	64.0–99.8	17.97	11.7–25.7	1.13	0.43
> − 0.5	76.92	46.2–95.0	21.87	15.1–30.0	0.98	1.05
> 0	69.23	38.6–90.9	22.66	15.7–30.9	0.90	1.36
> 0.5	61.54	31.6–86.1	29.69	21.9–38.4	0.88	1.30
> 2	64.54	31.6–86.1	52.34	43.3–61.2	1.29	0.73
> 2.5	53.85	25.1–80.8	66.41	57.5–74.5	1.60	0.70
> 3	38.46	13.9–68.4	70.31	61.6–78.1	1.30	0.88
> 3.5	30.77	9.1–61.4	73.44	64.9–80.9	1.16	0.94
> 4	23.08	5.0–53.8	78.91	70.8–85.6	1.09	0.97
> 4.5	15.38	1.9–45.4	81.25	73.4–87.6	0.82	1.04
> 5	0.00	0.0–24.7	87.50	80.5–92.7	0.00	1.14
> 8.5	0.00	0.0–24.7	100.0	97.2–100.0		1.00

*PCP Pneumocystis* pneumonia, *CI* confidence interval, *LR* likelihood ratio

All variables of the PCP score were compared between the PCP and the non-PCP groups (Table 5). There was no significant difference between the two groups. In addition, the results of univariable analysis (using logistic regressiion model) of variables in the PCP score also showed no significant difference between the two groups.

**Table 5:** Variables in the PCP score

	PCP (*n* = 13)	No-PCP (*n* = 128)	*P* value^a^	Odds Ratio (95% CI)
Age			0.613	
< 50 years	4 (30.8)	33 (25.8)		–
50–70 years	6 (46.2)	76 (59.4)		0.65 (0.17–2.69)
≥ 70 years	3 (23.1)	19 (14.8)		1.30 (0.24–6.53)
Lymphoproliferative disease	6 (46.2)	61 (47.7)	0.307	0.94 (0.29–2.98)
No prophylaxis	13 (100.0)	98 (76.6)	0.107	–
Duration between respiratory symptom onset and ICU admission			0.936	
0–3 days	4 (30.8)	38 (29.7)		–
3–5 days	2 (15.4)	25 (19.5)		0.76 (0.10–4.20)
> 5 days	7 (53.8)	65 (50.8)		1.02 (0.29–4.12)
Shock at ICU admission	5 (38.5)	37 (28.9)	0.690	1.54 (0.44–4.92)
Chest X-ray: not alveolar	5 (38.5)	45 (35.2)	1.000	1.39 (0.33–3.66)
Pleural effusion	3 (23.1)	41 (32.0)	0.727	1.08 (0.14–2.21)

Values are given as n (percentage)

*ICU* intensive care unit, *PCP P. jirovecii* pneumonia, *CI* confidence interval

^a^Data were compared using Fisher’s exact test

## Discussion

Over the last decade, there has been a substantial decline in PCP-related mortality rates among HIV-positive patients. Conversely, there is an increasing mortality rates associated with PCP in HIV-negative patients [14, 15]. In addition, a delay in anti-PCP treatment in these patients is associated with a higher mortality rate [13, 16]. This finding suggests that empiric therapy for PCP should be initiated in patients with high clinical suspicion for PCP. Unfortunately, there is no clinical tool that rapidly identifies patients at risk of PCP. Therefore, a high index of suspicion using patient history and clinical presentation, are key factors in early diagnosis of PCP [22]. However, the clinical picture varies individually as the general hallmarks (fever, shortness of breath and diffuse infiltrates) of this disease do not consistently occur, especially in its early course [23]. Therefore, clinical diagnosis is complicated because no combination of symptoms, signs, blood chemistries and/or radiographic findings is specific for PCP [24].

Recently, Azoulay et al. suggested a multivariable predictive model to improve the early diagnosis of PCP in haematology patients with ARF requiring ICU admission [18]. Variables included in the model were age, lymphoproliferative disease, anti-PCP prophylaxis, number of days between onset of respiratory symptoms and ICU admission, shock, chest radiograph pattern, and pleural effusion. Higher scores, were associated with lymphoproliferative disease, no anti-PCP prophylaxis, more than a three-day duration between onset of respiratory symptoms and ICU admission, and no alveolar pattern on radiography. Meanwhile lower scores were associated with those patients over 50 years of age, shock, and pleural effusion. Specificity for PCP was 88%, and the negative predictive value was 97%. Calibrations and discriminations were good (area under the curve, 0.80 in the derivation cohort and 0.83 in the validation cohort).

However, there are several questions regarding the variables used in the final model. Firstly, those patients over the age of 50 years were associated with lower scores meaning a lower risk of PCP. The authors described these points to be in line with older patients receiving less frequently high-dose chemotherapy or stem cell transplantation. These conditions put patients at high risk for PCP. However, the majority of haematologic malignancies are diagnosed in elderly patients and the decision to treat might not only be determined by age, but also by combining performance and frailty [25, 26]. In addition, previous reports showed an association between age > 60 years and pulmonary Pneumocystis colonisation, especially in patients with rheumatoid arthritis [27, 28]. Secondly, chest X-ray findings of PCP are non-specific and sometimes normal. In some cases, PCP presents bilateral, symmetric opacities in an interstitial or alveolar pattern on a chest X-ray. This is associated with an increased frequency of spontaneous pneumothorax [23, 29, 30]. Therefore, high resolution computed tomography (known to be the most reliable imaging technique for the detection and differential diagnosis of PCP), is recommended in immunocompromised patients, complementing either a negative or a vague chest X-ray [31].

Ideally, critically ill patients should be admitted in the ICU as soon as possible to receive the best appropriate care. However, delays in admission are common due to triage, diagnostic and logistic reasons [32–34]. Therefore, the duration between respiratory symptom onset and ICU admissions vary depending on each hospital’s policy and ICU bed availability. Our study also showed delayed ICU admissions (5 days versus 10 days in the PCP group). In addition, PCP patients presented more shock (22.4% versus 38.5% in the PCP group) and pleural effusion (5.2% versus 23.1%) in our cohort which are inconsistent with the PCP scores in Azoulay et al. These results are explained by the delayed ICU admission in our cohort.

To the best of our knowledge, this is the first external validation of the PCP score, with varying predictive results in haematology patients from the Korean cohort. However, there were several potential limitations to our study. Firstly, although the sample size was larger than a priori (which was adequately powered to obtain the significant of an area under ROC curve of 0.8), it was relatively small compared to the original study. However, post hoc power analysis revealed that the observed power was 96.4% with a 9.2% prevalence of PCP in our cohort. Thus, the results are adequately powered to rule out the possibility of false-negative findings. Secondly, the diagnosis of PCP was confirmed by the identification of the organism in BAL fluid or lung tissue only. The diagnostic strategy for PCP now usually combines non-invasive diagnostic tests and PCR testing of BAL fluid. However, quantitative PCR was not yet available in Korea. Therefore, patients with positive PCR related to colonisation were considered as not having PCP in this study.

## Conclusion

From the analysis of the two cohorts from designated haematology centres in Korea, the PCP score were not useful to predict the risk of PCP in haematology patients. Further prospective studies are needed before the score can be implemented into routine clinical practice for the early diagnosis of PCP in haematology patients.

## Data Availability

The data that support the findings of this study are available on request from the corresponding author. The data are not publicly available due to privacy or ethical restrictions.

## Abbreviations

ARF: Acute respiratory failure
BAL: Bronchoalveolar lavage
CI: Confidence interval
HIV: Human immunodeficiency virus
ICU: Intensive care unit
IQR: Interquartile range
LR: Likelihood ratio
OR: Odds ratio
PCP: *Pneumocystis* pneumonia
SOFA: Sequential Organ Failure Assessment
TBLB: Transbronchial lung biopsy
